# IGESS: A CO-CREATIVE INTERGENERATIONAL STRATEGY FOR ENHANCING POSITIVE INTERGENERATIONAL OUTCOMES

**DOI:** 10.1093/geroni/igae098.4281

**Published:** 2024-12-31

**Authors:** Alice C Wong, Daniel W L Lai, Alison Y T Ou, Xue Bai

**Affiliations:** Hong Kong Baptist University, Hong Kong, Hong Kong, China (People’s Republic); Hong Kong Baptist University, Hong Kong, Hong Kong, China (People’s Republic); Hong Kong Baptist University, Hong Kong, Hong Kong, China (People’s Republic); The Hong Kong Polytechnic University, Hong Kong, Hong Kong, China (People’s Republic)

## Abstract

Intergenerational interaction is crucial for promoting social cohesion and mutual understanding. The Inter-Generational Engagement in Secondary Schools (i-GESS) program was created to engage older adults and university students in co-creating learning activities for students in secondary schools to enhance positive intergenerational interactions, attitude, knowledge, and skills. This study examined the changes using a quasi-experimental pre-post survey design in 129 older adults (age 60+), 110 university students (age 18 to 22), and 470 secondary school students (age 13 - 16). The results demonstrated substantial improvements in attitudes towards different age groups, as measured by the 34-item Kogan’s scale. Secondary school students showed a significant 13.7% improvement in attitudes towards older adults, while university students and older adults noted increases of 4.2% and 5%, respectively, in their views towards youth. Additionally, self-rated communication skills saw notable gains, with secondary school students and university students recording increases of 7.9% and 9.5%, respectively. Trust in communication also increased substantially, evidenced by a 12.1% rise among secondary school students and a 5% improvement among older adults. The most pronounced enhancements were in the attitudes of secondary school students towards older adults and the communication skills of university students, indicating major impacts in these areas. The i-GESS initiative successfully bridged generational divides, fostering meaningful relationships across age groups via the application of the co-creation strategy. The positive outcomes further reinforce the benefits of this intergenerational approach for enhancing mutual cooperation and respect.

